# Gum chewing and bowel function after Caesarean section under spinal anesthesia

**DOI:** 10.12669/pjms.345.15772

**Published:** 2018

**Authors:** Ozlem Akalpler, Hulya Okumus

**Affiliations:** 1 Ozlem Akalpler, M.Sc. Department of Obstetrics, Women’s Health and Gynecology, Faculty of Nursing, Near East University, Nicosia, Cyprus; 2 Prof. Dr. Hulya Okumus, Department of Obstetrics, Women’s Health and Gynecology, Faculty of Nursing, 9 Eylul University, Izmir, Turkey

**Keywords:** Cesarean section, Gum chewing, Postoperative care, Spinal anesthesia

## Abstract

**Objective:**

To determine the effect of gum chewing on bowel function postoperatively in women after cesarean delivery under spinal anesthesia.

**Methods:**

This semi-experimental study was conducted at Near East University Hospital Obstetrics and Gynecology Clinic in Turkish Republic of Northern Cyprus with 45 women in both the +experimental and control groups, between October 2016 and June 2017. The women in the experimental group chewed gum two hours after surgery until gut sounds were heard and nutrition was given orally.

**Results:**

The mean age of the women was 30.20 ± 4.88 in the experimental group and 30.85 ± 4.47 in the control group. In the control group, the mean time of flatulation was 26.33 ± 7.54 hours, whereas the mean time was 13.44 ± 6.56 hours in the experimental group. The mean duration to the hearing of intestinal sounds was 16.35 ± 5.20 hours in the control group and 12.62 ± 7.73 hours in the experimental group.

**Conclusion:**

The results of the present study indicate that sugar free gum chewing in the post-operative period which is cesarean section under spinal anesthesia shortened the duration of the intestinal movement, the time of first flatulation, and discharge time.

## INTRODUCTION

According to 2015 World Health Organization (WHO) data, the rates of cesarean delivery are 33% in the United States, 38% in Italy, 56% in Brazil, 46% in Mexico, 33% in Switzerland, and 32% in Germany.^1^ In the Turkish Republic of Northern Cyprus (TRNC), the Ministry of Health states the rate of cesarean deliveries is 53.1% in state hospitals. There is no data for private hospitals.^2^

Small intestine activity after abdominal operations returns to normal function within a few hours, gastric activity returns to normal within 24-48 hours, and colon activity returns to normal within 48-72 hours.^3^,^4^ Due to the delayed motility of the gastrointestinal system in the postoperative period, gas and secretions accumulate in the stomach and small and large intestines, which causes abdominal distension, nausea, vomiting, and pain, all of which negatively affect the comfort level of the patients.^5^ Chewing gum activates the cephalic vagal reflex and can be used to stimulate bowel function in the postoperative period. Chewing gum stimulates the digestive cephalic phase by imitating eating and is considered a virtual diet.^6^-^9^

Early initiation of intestinal motility after abdominal surgery is highlighted in the “Enhanced Recovery After Surgery” (ERAS) protocol, also known as the multidisciplinary approach.^10^ In this protocol, it was noted that for the prevention of postoperative ileus development, having the patient chew gum has moderate evidence. Approaches suggested in the ERAS protocol can also be applied after cesarean section, which speeds up the recovery process in the postoperative period.^11^ Late onset of bowel movements after cesarean section with spinal anesthesia causes the mother to feel uncomfortable and to suffer difficulties in breast feeding due to pain and distension. It also causes problems such as late discharge, hospital infections, and high costs for the hospitals and families.

In the literature, there are studies in which gum chewing accelerates the healing process and shortens the length of stay in the hospital by increasing intestinal motility after abdominal surgery.^6^-^9^

Harma et al. in a study on patients after cesarean section surgery showed that chewing gum shortened the time first intestinal movements started.^12^ In Ledari study, on 100 women candidates for cesarean section with local anesthesia (spinal), in gum-chewing group, the first bowel sounds in post operative period after C. Section was significantly shorter compared to the control group (p=0.016).^13^

This study aim was to determine the effect of sugar-free gum chewing on bowel function postoperatively in women following cesarean delivery under spinal anesthesia.

## METHODS

This semi-experimental study was conducted at Near East University Hospital Obstetrics and Gynecology Clinic in Turkish Republic of Northern Cyprus with 45 women in both the experimental and control groups, between October 2016 and June 2017. The sample consisted of patients with elective or emergency cesarean section on a volunteer basis. The women were over 18 years old, received spinal anesthesia, did not have any systemic or chronic disease, and had not undergone tooth or jaw surgery or have a history of dental disease. In the study, the conclusions of Harma et al.^12^ and Ledari et al.^13^ were utilized to calculate the sample size. G*Power software (Version 3.1.7) was used for calculations. Power analysis was calculated to be 90% and the error rate to be 5%. The number of samples according to this calculation was experimental (n: 45) and control (n: 45).

Women in the experimental group were asked to chew commercially available soft, unsweetened, easily chewable gum, containing no aspartame, sorbitol, or xylitol from the second hour of the postoperative period until oral feeding. In the postoperative period, the patient chewed gum at least three times for a minimum of 15 minutes and maximum of 30 minutes during the 2nd, 4th, and 6th hours and they did not eat anything other than gum for the first six hours. In the control group, no application other than standard post-operative care was carried out. The women included in the study were informed about the study and their written consent was obtained. Ethical approval was obtained from the institution where the study was carried out (YDU/2015/35-251).

### Data Collection

The data were collected using two forms.

***1. Socio-Demographic Information:*** This form consisted of seven questions regarding age, educational status, profession, and working status and five questions regarding obstetric history. The researcher completed the socio-demographic information from using face-to-face interviews with the pregnant women before surgery.

***2. Bowel Function Evaluation Form:*** In the bowel function evaluation form prepared for the experimental group, there were four questions regarding the time the patient left surgery, gum chewing times and durations, intestinal sounds, and flatulation time. In the bowel function evaluation form prepared for the control group, there were three questions regarding the time the patient left surgery, intestinal sounds, and flatulation time.

### Research Hypotheses

**H1:** There is a difference between the time of first flatulation for the patients in the experimental and control groups.

**H2:** There is a difference between the time of first bowel sounds for the patients in the experimental and control groups.

**H3:** There is a difference between the patient discharge times in the experimental and control groups.

### Analysis

Arithmetic mean, standard deviation, median, and minimum-maximum values were determined as descriptive statistics for quantitative data. Descriptive statistics for qualitative variables were shown as numbers and percentages. For continuous data, the Mann-Whitney U test was applied for comparisons between the two independent groups. The Kruskal-Wallis test was applied to compare continuous data among multiple groups. For hypothesis tests related to categorical variables, Pearson’s Chi Square and Fisher’s exact Chi Square tests were applied. Statistical power was 90% and the error rate was 5%. The significance level for all studies was accepted as 0.05. Post-hoc study conclusion statistical power analysis revealed 98.99% for hypothesis H1, 100% for hypothesis H2, and 59.08% for hypothesis H3. Statistical evaluation of the data was performed using SPSS (Version 17.0) software.

## RESULTS

The experimental and control groups were statistically homogeneous in terms of age, educational status, occupation, working status, previous surgery, continuous drug use, and normal bowel habits (Table-I).

**Table-I:** Distribution of women in experimental and control groups by socio-demographic characteristics.

Descriptive Characteristics	Control Group (n: 45)		Experimental Group (n: 45)		Total (n: 90)			

	n	%	n	%	n	%	P*
***Age***							
25 years and less	7	15.6	7	15.6	14	15.6	
26-30 years	14	31.1	16	35.6	30	33.3	0.896
31 years and more	24	53.3	22	48.9	46	51.1	
***Educational Status***							
High School and lower	13	28.9	17	37.8	30	33.3	
University	32	71.1	28	62.2	60	66.7	0.371
***Occupation***							
Housewife	12	26.7	17	37.8	29	32.2	0.472
Officer	6	13.3	6	13.3	12	13.3		
Worker	23	51.1	16	35.6	39	43.3	
Freelancer	4	8.9	6	13.3	10	11.1		
***Working status***							
Working	26	57.8	20	44.4	46	51.1	0.206
Not working	19	42.2	25	55.6	44	48.9	
***Previous surgeries***							
Yes	26	57.8	28	62.2	54	60.0	0.667
No	19	42.2	17	37.8	36	40.0	
***Permanent drug use***							
Yes	7	15.6	2	4.4	9	10.0	**0.157
No	38	84.4	43	95.6	81	90.0	
***Normal bowel habits***							
Everyday	22	48.9	24	53.3	46	51.1	0.697
Every two days	14	31.1	15	33.3	29	32.2	
Every three to five days	9	20.0	6	13.3	15	16.7	

* Pearson Chi-square test, (**) Fisher’s exact Chi-square test.

As a result of examining the bowel functions after the cesarean section of the women in the study group, the mean time for women to flatulate in the experimental group was 13.44 ± 6.56 hours and for women in the control group was 26.33 ± 7.54 hours. The shortened duration until flatulation in the experimental group was statistically significant (p<0.001) (Table-II). The mean duration leading to the hearing of intestinal sounds was 16.35 ± 5.20 hours in the control group and 12.62 ± 7.73 hours in the experimental group. The time of hearing the bowel movements in the experimental group was statistically significant (p<0.001) (Table-II).

**Table-II:** Distribution of women in the experimental and control groups according to intestinal functions after cesarean section.

	Control Group (n: 45)		Experimental Group (n: 45)		p

	Avg.+SD	Median (MinMax)	Avg.+SD	Median (MinMax)	
Time to hear intestinal sounds (h)	16.35±5.20	18.00 (4.0024.00)	12.62±7.73	15.00 (2.0027.00)	0.001
Time to first flatus (h)	26.33±7.54	26.00 (10.0046.00)	13.44±6.56	18.00 (4.0024.00)	0.001
Time to discharge (h)	49.11±1.62	48.00 (48.0054.00)	47.17±1.51	48.00 (44.0048.00)	0.036

According to the findings obtained in this study, gum chewing shortened the duration of the intestinal movements in the post-operative period, the time of first flatulation, and discharge. These results support the hypotheses H1, H2, and H3.

## DISCUSSION

Problems such as constipation, postoperative ileus, and abdominal distension may be seen in the gastrointestinal system due to the effect of anesthesia after abdominal surgery.^5^ For early return of bowel movements after caesarean section surgeries, applications such as oral hydration, early feeding^14^, and gum chewing^9^,^13^,^15^-^21^ are recommended in the postoperative period.

The mean time for bowel sounds in women following cesarean delivery with spinal anesthesia included in this study was 12.62 ± 7.73 hours in the experimental group and 16.35 ± 5.20 hours in the control group. The fact that bowel sounds started four hours earlier in the experimental group was significant. A four hour difference for a woman who has delivered via caesarean section is a positive result in terms of comfort and prevention of complications.

The study of Abd-El Maeboud et al.^22^ with 200 women after cesarean delivery under general anesthesia in Egypt revealed that the group that chewed gum experienced bowel sounds 10.9 hours after surgery and the control group 15.6 hours after surgery. Shang et al.^23^ conducted a study in China (n:388) to determine the effect of chewing gum on postoperative bowel function in spinal anesthesia patients who delivered via cesarean section and they revealed that bowel sounds started 18.2 hours after surgery in the experimental group and 23.2 hours after surgery in the control group. In the randomized controlled study of Ledari et al.^13^ with 100 women, the first bowel sounds after cesarean section were 21.9 hours in the gum chewing group and 26.1 hours in the control group. The five-hour difference between the groups is important for patient comfort and discharge. In this study, the bowel sounds started four hours earlier in the experimental group, similar to the above studies (Table-II). Kafali et al.^24^ conducted a study with 150 women in order to evaluate the effect of chewing gum after cesarean section on postoperative bowel activity and bowel sounds were significantly shorter in the study group (mean 5.9 hours) than in the control group (mean 6.7 hours) (p<0.01). The results obtained are similar to the results of this study. This similarity suggests that chewing gum in the postoperative period accelerates intestinal motility, early bowel sounds, patient comfort, early discharge, and cost-effectiveness.

In this study, women in the gum-chewing group flatulated after an average of 13.44 ± 6.56 hours and those in the control group after 26.33 ± 7.54 hours (Table-II). In the study of Kafali et al.^24^ with women who delivered by caesarean section (n:150), women in the experimental group flatulated 22.4 hours after surgery and women in the control group flatulated 31 hours after surgery. In the experimental study of Shang et al.^23^, the mean flatulation time for the women in the gum chewing group was 5.3 hours earlier than in the control group. This study achieved similar results. The average 13 hour difference in flatulation time between the experimental group and the control group ensured that the patients in the experimental group felt better. Chewing gum stimulates intestinal motility by mimicking eating. It is thought that the earlier flatulation for the gum chewing group after surgery is due to this effect. This result is a positive finding of gum chewing on bowel motility.

Several meta-analysis studies have reported that chewing gum enhances bowel movements after gastrointestinal surgery.^25^,^26^ The results obtained from this study can contribute to future meta-analysis studies. Some studies in the literature state that chewing gum increases intestinal motility after abdominal surgery, accelerates the healing process, and shortens the time to discharge from the hospital.^12^,^17^,^23^,^24^ In this study, the women who chewed gum had shorter discharge times (47.17 ± 1.51 hours) than those in the control group (49.11 ± 1.62 hours). In the study of Abd-El-Maeboud et al.^22^, the women in the experimental group were discharged after 40.8 hours and the women in the control group after 50.5 hours (p<0.05). According to the results obtained from our study, the time of discharge after surgery in the chewing gum group was two hours earlier. Although this finding was not statistically significant, it is clinically important for maternal and infant health.

## CONCLUSION

The current study was performed to investigate the effect of gum chewing on the return of intestinal function in women with cesarean section under the spinal anesthesia, so that a positive step can be taken toward diminishing their problems in fields of timely and early prevention of ileus. According to the findings obtained in this study, gum chewing shortened the duration of the intestinal movements, first flatus, time to hear intestinal sounds and time to discharge in patients undergoing cesarean section under the spinal anesthesia in the post-operative period. It can be added to post-caesarean care without any concern on early post-operation feeding as a low-cost, safe and tolerable treatment in early intestinal stimulation to reduce ileus associated complications.

### Authors’ Contribution

**OA, HO** conceived, designed and did statistical analysis & editing of manuscript.

**OA, HO** did data collection and manuscript writing.

**OA, HO** did review and final approval of manuscript.
